# Psychosocial Factors Predict the Level of Substance Craving of People with Drug Addiction: A Machine Learning Approach

**DOI:** 10.3390/ijerph182212175

**Published:** 2021-11-19

**Authors:** Hua Gong, Chuyin Xie, Chengfu Yu, Nan Sun, Hong Lu, Ying Xie

**Affiliations:** 1 Department of Psychology, School of Education, Guangzhou University, Guangzhou 510006, China; 2111908034@e.gzhu.edu.cn (H.G.); crazy.xiaoxun@163.com (C.X.); yuchengfu@gzhu.edu.cn (C.Y.); nansun2019@gzhu.edu.cn (N.S.); 2 Research Center of Adolescent Psychology and Behavior, Guangzhou University, Guangzhou 510006, China; 3 Center for Brain and Cognitive Sciences, Guangzhou University, Guangzhou 510006, China; 4 Department of Sociology, School of Public Administration, Guangzhou University, Guangzhou 510006, China

**Keywords:** substance craving, life events, aggression behavior, impulsivity, gradient boosting method

## Abstract

This study aimed to explore which factors had a greater impact on substance craving in people with substance use and the direction of the impact. A total of 895 male substance users completed questionnaires regarding substance craving, psychological security, positive psychological capital, interpersonal trust, alexithymia, impulsivity, parental conflict, aggression behavior, life events, family intimacy, and deviant peers. Calculating the factor importance by gradient boosting method (GBM), found that the psychosocial factors that had a greater impact on substance craving were, in order, life events, aggression behavior, positive psychological capital, interpersonal trust, psychological security, impulsivity, alexithymia, family intimacy, parental conflict, and deviant peers. Correlation analysis showed that life events, positive psychological capital, interpersonal trust, psychological security, and family intimacy negatively predicted substance craving, while aggression behavior, impulsivity, alexithymia, parental conflict, and deviant peers positively predicted substance cravings. These findings have important implications for the prevention and intervention of substance craving behavior among substance users.

## 1. Introduction

Substance abuse not only causes physiological and psychological harm for users and their families but also serious consequences for society [1]. According to *the World Drug Report 2021*, over the past year, around 275 million people have used drugs, up by 22 percent from 2010. By 2030, demographic factors project the number of people using drugs to rise by 11 percent around the world [2]. Relapse is one of the most common behaviors of substance users [3,4,5], and substance craving is one of the determinants of relapse [6,7,8,9], it refers to the strong urge for addictive substances produced by substance users, which forces individuals to take substances to achieve psychological satisfaction to avoid withdrawal reactions [10]. Given the social and economic impact of substance craving, it is crucial improving the comprehension of the factors that may help reduce the craving behavior of substance users.

According to the holistic-integrated mode [11], individual behavior is formed and developed in the functional and dynamic interaction between external and internal factors [12]. Specifically, it is important to understand which external and internal factors may influence substance craving behaviors [13]. In terms of internal factors, it mainly focuses on personality [14,15,16], emotion [17,18], and withdrawal symptoms [19,20]. For example, a study found that callousness can modulate the substance craving response of high-risk juvenile delinquents [21]. The research on external factors mainly include physical stimuli related to substance [22,23], stress [4,8], social support [24], peers [25,26], and so on. For instance, Preston and his colleagues [27] found that the craving for substance is greater with the combination of substance cues and stress than with either alone in substance-dependent patients.

In summary, although there are many studies on the factors affecting substance craving, there is a lack of systematic assessment of the importance of psychosocial variables in substance craving. Based on this, we intend to use the Gradient Boosting Method (GBM) in machine learning to calculate the weight of each variable (highly correlated with substance craving) and determine the factors with higher importance via preliminary screening and sorting. Compared with traditional data mining techniques, this method has higher prediction accuracy and efficiency [28,29,30], and its effect has been verified in various fields [31,32,33]. Nevertheless, in the field of substance craving, the analysis of data mostly relies on regression analysis, variance analysis, and other methods [34,35]. Thus, the introduction of GBM with high prediction accuracy not only helps to expand the scope of substance craving research methods but also provides a reference for the subsequent development of more effective and reasonable intervention schemes (i.e., prioritizing variables of higher importance). In this study, we screened out ten factors (both internal and external) that may have an important influence on substance craving from previous studies, and analyzed their importance by GBM.

There are many studies to explore the mechanism of substance craving from the physiological aspect (such as [36]), however, the impact of psychological and social factors on substance craving is unclear. According to the biopsychosocial (BPS) model [37], psychological and social variables also play a crucial role in shaping individual behaviors. Due to there being few studies directly exploring the relationship between substance craving and psychological or social variables, we selected some psychological or social variables closely related to substance use, analyzed their importance, and proposed our hypotheses:

**Hypothesis** **H1.** *Psychological variables have a greater impact on substance craving than social variables.*

**Hypothesis** **H2a.** *Among psychological variables, positive psychological capital, interpersonal trust and psychological security negatively predicted substance craving; aggressive behavior, impulsivity and alexithymia positively predicted substance craving.*

**Hypothesis** **H2b.** *Among social variables, family intimacy negatively predicted substance craving; life events, parental conflict, and deviant peers positively predicted substance craving.*

## 2. Materials and Methods

### 2.1. Participants

We have recruited 895 males with substance use disorder (*M_age_* = 37.87 years, *SD* = 8.40 years) from Zengcheng Compulsory Isolated Detoxification Center (ZCIDC) by using cluster sampling. Among the participants, 69.7% had completed junior high school or higher, 54.2% were employed, 41.5% and had never married. The substance-related data show that 49.2% used heroin, 39.3% used cocaine, and the rest used polysubstance; the average age of onset of use was 24.60 years (*SD* = 7.02). Before completing the survey, all the participants gave written informed consent. All of them can understand the terms in the questionnaire. Participants who left more than 30% of items uncompleted were not included in the final analysis. Missing values of the data were replaced with averages. This study was approved by the Ethics Committee of Guangzhou University. The protocol number is GZHU2020012.

### 2.2. Measures

*Drug craving scale(DCS)*: The DCS was compiled based on classical conditioning and operant conditioning theory, which includes 34 items, and measures five dimensions of substance craving, including (1) prizing substance craving, the positive affective experience elicited by reward effect and positive reinforcement (8 items, e.g., “I often recall how comfortable I felt when used substances”); (2) reflecting substance craving, the reflexive subjective experiences evoked by someone or something associated with substances in the mind (5 items, e.g., “I want to use substances when recall the place I bought it”); (3) social substance craving, the impact of social variables on substance users (7 items, e.g., “If I get out and don’t have a job, I might relapse”); (4) negative substance craving, the craving aroused by negative emotions (5 items, e.g., “When suffered setbacks, I think everything gone be fine after used substances”); and (5) eliminating substance craving, to eliminate the discomfort on physical and mental (9 items, e.g., “If I have insomnia, I want to use substances”) [38]. Participants were required to respond to each item from 1 (strongly disagree) to 7 (strongly agree). Cronbach’s α was 0.98 in this study.

*Security Questionnaire (SQ)*: The SQ is a 16-item questionnaire that measures the psychological security of persons with substance use (e.g., “No matter what others say, I feel useless”) [39]. Participants were required to respond to each item from 1(strongly agree) to 5 (strongly disagree). Cronbach’s α was 0.88 in this study.

*Positive psychological capital (PPQ)*: We adopted the 26-item from Zhang et al. [40] to measure positive psychological capital (e.g., “I am confident of my ability”). Then participants should respond to each item from 1(strongly disagree) to 7(strongly agree). Cronbach’s α was 0.92 in this study.

*Alexithymia*: We measured alexithymia by using the Toronto Alexithymia Scale (TAS-20) [41]. This scale contains three dimensions, one focusing on emotion recognition, and others focusing on emotion description and extroversion thinking (e.g., “I experiencing some unrecognized feeling”). Participants were required to respond to each item from 1(strongly agree) to 5(strongly disagree). Cronbach’s α was 0.72 in this study.

*Impulsivity*: This scale included 20 items that measure five dimensions of impulsivity, including negative urgency, positive urgency, programmatic, perseverance, and sensation seeking (e.g., “I am very adventurous”) [42]. Each item was rated on a scale from 1(strongly disagree) to 4(strongly agree). Cronbach’s α was 0.74 in this study.

*Parental conflict*: This scale including 7-item that adapted from the Children’s Perception of Inter-parental Conflict Scale (CPIC) (e.g., “my parents always argue”) [43]. One sample was that ‘My parents always argue’. Each item was rated on a scale from 1(strongly disagree) to 5(strongly agree). Cronbach’s α was 0.75 in this study.

*Interpersonal trust (ITS)*: We used the Interpersonal Trust Scales to assess participants’ interpersonal trust which includes 25 items (e.g., “parents usually can be relied upon to keep their promises”) [44]. Participants were required to rate on a 5-point Likert scale. The 5-point scale ranged from 1(strongly disagree) to 5(strongly agree). Cronbach’s α was 0.84 in this study.

*Aggression behavior*: We adopted the Buss-Warren Aggression Questionnaire-Revised in China (BWAQ-RC) [45] to measure the level of aggressive behavior of persons with addictions, which included 34 items (e.g., “I find it very hard to control my temper”). Then participants should respond to each item from 1 (not like me at all) to 5 (very much like me). Cronbach’s α was 0.94 in this study.

*Life EventScale(LES)*: The scale measures life events from three aspects: family life, work and study, social interaction, and others, including 48 items (e.g., “I don’t get along with my colleagues and neighbors”) [46]. Participants were asked to rate the degree to which the event affected their mental state (experiencing nervousness, stress, excitement, or distress, etc.,) from 1 (no effect) to 5 (extremely heavy). Cronbach’s α was 0.97 in this study.

*Family intimacy*: This scale included 9 items adapted from the Family Environment Scale-Chinese version (FES-CV) (e.g., “our family members always give each other the most help and support”) [47]. Participants were required to respond to each item from 1 (never) to 5 (always). Cronbach’s α was 0.85 in this study.

*Deviant peers*: We adapted a 16-item scale to measure deviant peers (e.g., “how many of your friends have been in fights”) [48]. Participants were asked to report the number of companions who engaged in 16 types of bad behavior, such as fighting, absenteeism, or truancy. Using a 5-point scale. Cronbach’s α was 0.91 in this study.

### 2.3. Procedure

The test was conducted in April 2018, and the experimenters were postgraduate students majoring in psychology who had received training. After obtaining the participants’ consent, the assistant took them to the lab, leaving the participants to complete the experiment alone. Before the test, the guide was announced by the experimenter, the participants were asked to fill in the questionnaire according to their real situation, and emphasizing the confidentiality of the test results. The test was completed at one time, and the test time was about 30 min.

### 2.4. Data Analyses

With the rapid development of deep learning methods, machine learning has become a major tool in the field of statistics and made great achievements [49]. Machine learning is increasing in popularity in various fields. It has also been applied to psychology in recent years. Many studies utilized machine learning via substantial data from online personal databases to predict one’s psychological characteristics [50,51,52]. For example, Bleidorn and Hopwood [53] carried out a comprehensive review of the application of machine learning in the improvement of human personality. Moreover, it can also be used to predict human behaviors. Srividya and her colleagues [54] used machine learning algorithms to predict the onset of mental illness. In this study, machine learning was used to predict the possible effects of potential variables on substance craving among people with substance use.

#### 2.4.1. GBM (Gradient Boosting Method)

Gradient boosting method (GBM) is a prediction model in the form of an ensemble of weak prediction models for classification issues. By optimizing an arbitrary differentiable loss function on an appropriate cost function [55], GBM is a tree ensemble model constructed by a series of classification and regression trees that attempt to define and optimize an objective function. In the current study, we utilize the gradient boosting regression tree (GBRT) that considers additive models described as the following equation:

(1) $$F\left(x\right)=\overset{M}{\underset{m=1}{\sum }}\gamma_{m}h_{m}\left(x\right)$$

where $h_{m}\left(x\right)$ are the basic functions that are usually called weak learners in the context of boosting. Subsequently, GBRT establishes the additive model in a forward fashion:

(2) $$F_{m}\left(x\right)=F_{m-1}\left(x\right)+\gamma_{m}h_{m}\left(x\right)$$

At each stage of the decision tree, $h_{m}\left(x\right)$ is chosen to minimize the loss function L given the current model $F_{m-1}$ and its fit $F_{m-1}\left(x_{i}\right)$

(3) $$F_{m}\left(x\right)=F_{m-1}\left(x\right)+\underset{h}{\min}\overset{n}{\underset{i=1}{\sum }}L\left(y_{i},F_{m-1}\left(x_{i}\right)-h\left(x\right)\right)$$

The initial model $F_{0}$ is problem-specific, for example, one usually chooses the mean of target values. GBM endeavors to solve this problem of minimization numerically via the steepest descent. The steepest descent direction is the negative gradient of the loss function evaluated at the current model $F_{m-1}$, which can be calculated for any differentiable loss function:

(4) $$F_{m}\left(x\right)=F_{m-1}\left(x\right)+\gamma_{m}\overset{n}{\underset{i=1}{\sum }}\nabla_{F}L\left(y_{i},F_{m-1}\left(x_{i}\right)\right)$$

where the step length $\gamma_{m}$ is chosen using a line search:

(5) $$\gamma_{m}=\arg\underset{h}{\min}\overset{n}{\underset{i=1}{\sum }}L\left(y_{i},F_{m-1}\left(x_{i}\right)-\gamma \frac{\partial L\left(y_{i},F_{m-1}\left(x_{i}\right)\right)}{\partial F_{m-1}\left(x_{i}\right)}\right)$$

#### 2.4.2. Factor Importance

The relative importance of the feature is assessed according to the predictive contribution of the target variable [56]. We utilized five-fold cross-validation, or out-of-sample testing techniques, to estimate the accuracy of a predictive model. Cross-validation divides the data into the given number of subsets, executes the algorithm on the training dataset, and then verifies the performance on the testing dataset. The validation results are averaged over five rounds to provide an estimate of the model’s predictive performance.

## 3. Results

### 3.1. The Degree of Influence of Different Variables on Substance Craving

**Figure 1:**
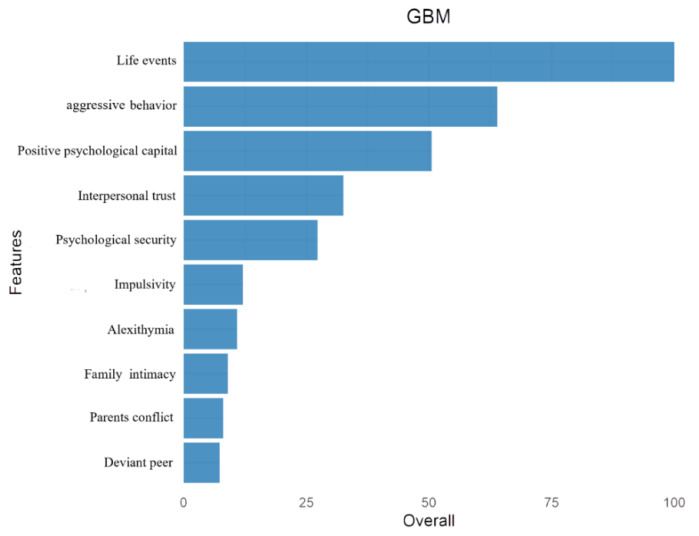
The influencing factor model is based on GBM.

To assess the possible influences of variables on substance craving, the GBM was employed to make a preliminary prediction. As shown in Figure 1, we can easily find that the first five important factors are life events, aggressive behavior, positive psychological capital, interpersonal trust, and psychological security. The second five factors that have an impact on substance craving are impulsivity, alexithymia, family intimacy, parent conflict, and deviant peer. Surprisingly, results show that social variables have a greater influence on substance craving than psychological variables, especially life events play the most important role in predicting substance craving, which is not consistent with Hypothesis H1. Although Figure 1 shows the importance ranking of those variables, it does not tell us the positive or negative effects on substance craving. Therefore, further analysis should be conducted to get more precise and valid results that may be useful for predicting substance craving behavior in people with substance use.

### 3.2. The Relationship between Psychological Variables and Substance Craving

**Figure 2:**
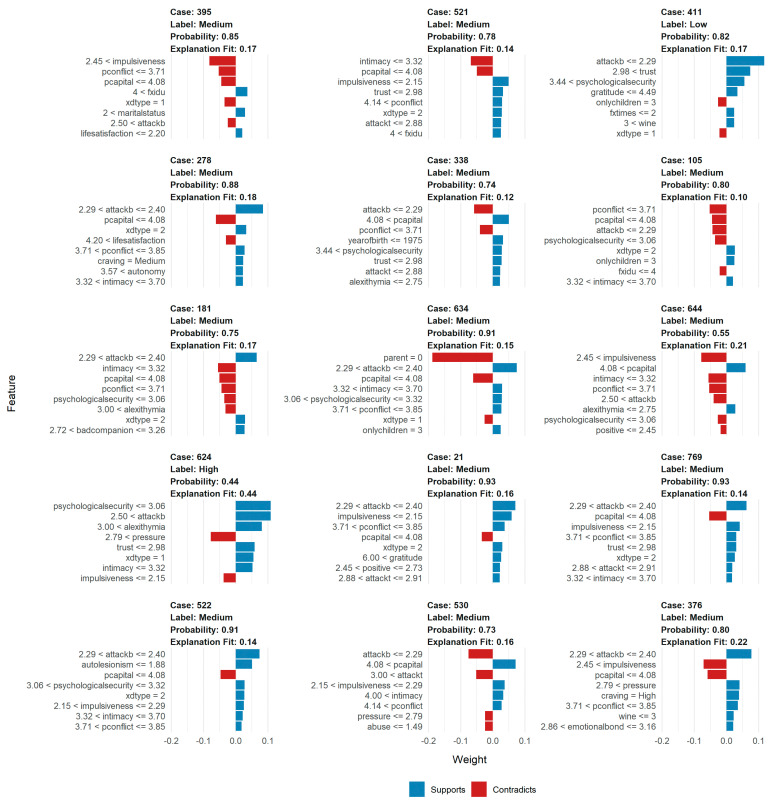
Correlations between factors and substance craving.

From Figure 2, a preliminary analysis of the relationship between the level of substance craving and the score of aggressive behavior, positive psychological capital, interpersonal trust, psychological security, impulsivity, and alexithymia was conducted. Label = High, Medium, or Low means the level of substance craving. The bar diagrams in blue on the right side support the “Label” while the left ones contradict the “Label”. The result was supported Hypothesis H2a, it predicts a high level of substance craving when the psychological security score drops below 3.06, the level of alexithymia is higher than 3.00, and the score of positive psychological capital is below 4.08. Conversely, when the score of impulsivity is lower than 2.15, the score of aggressive behavior drops below 2.29, and the score of interpersonal trust is higher than 2.98, which indicates a low level of substance craving.

### 3.3. The Relationship between Social Variables Substance Craving

From Figure 2, a preliminary analysis of the relationship between the level of substance craving and the score of life events, family intimacy, parental conflict, and deviant peer was conducted. The result was partially supported Hypothesis H2b, it forecasts a low level of substance craving when the life events score higher than 2.79 and the score of parental conflict is below 3.71. On the contrary, when the score of family intimacy is lower than 3.32 and the score of deviant peers is higher than 2.72, means a high level of substance craving. The hypothesis of life events has not been proved, in this study, life events have a negative impact on substance craving, which is not consistent with the previous studies [57,58], and we discussed it in the next part.

## 4. Discussion

Little research has simultaneously investigated the relationship between numerous potential influencing variables and substance craving, and the extent to which these variables affect substance craving. This study explored the effects of life events, impulsivity, alexithymia, and other variables on substance craving in persons with addictions from the perspective of psychological and social factors. The results showed that among the social factors, life events had the greatest influence on substance craving, while aggressive behavior had the greatest influence on substance craving among the psychological factors. These findings contribute to a better understanding of the positive and negative effects of different variables on substance craving from a comprehensive perspective and provide evidence for the prevention and intervention of substance craving behavior.

### 4.1. The Impact of Social Variables on Substance Craving

First of all, our findings suggested that life events were negatively associated with substance craving, whereas previous studies have shown that life events have a positive impact on substance craving. We argued that maybe the properties of life events lead to the difference. In detail, previous studies have mainly focused on stressful life events [57], while our study focuses on both positive and stressful life events. That is to say, the substance craving might be alleviated if substance users experienced more positive life events. Therefore, the influence of different types of life events, especially positive life events (such as getting married, becoming a parent), on substance craving could be further explored in the future.

Similarly, GBM shows that substance craving was positively correlated with parental conflict, and deviant peers, negatively correlated with family intimacy. However, many studies have focused on the influence of those variables on substance use [59,60,61], neglecting their relationship with substance craving. According to the Social Identity Model of Addiction (SIMOR) [62], social factors play a crucial role in the addiction of substance users, and the social factors that have an important influence on substance craving may differ at different stages of recovery [63], thus, it is necessary to explore the relationship between social variables and substance craving. In recent years, research on deviant peers has found that spending time with deviant peers can boost craving for substances [25,26]. Unfortunately, research on substance craving and parental conflict or family intimacy are scarce. Although these factors are not as significant as life events for substance craving, it does not mean that they should be ignored [64]. Instead, subsequent research could further explore their relationship (such as mediating or moderating effect) with substance craving, which could help make better recovery plans for substance users. Finally, although Hypothesis H1 is not supported and Hypothesis H2b is partially supported, its implications that should pay attention to social factors is important for alleviating craving among substance users.

### 4.2. The Impact of Psychological Variables on Substance Craving

Our study found that aggressive behavior, impulsivity, and alexithymia were positively correlated with substance craving; positive psychological capital, interpersonal trust, and psychological security were negatively correlated with substance craving, which is consistent with Hypothesis H2a. It is worth mentioning that impulsivity and alexithymia have been studied extensively in substance craving [65,66], while other variables have been poorly explored.

First, impulsivity and alexithymia have been proved that could promote substance craving [20,67]. A study of patients with alcohol use disorder (AUD) found that AUD patients with a predisposition for rash impulsiveness are more vulnerable to alcohol craving, and subsequently, poorer treatment outcomes [68]. As for the mechanism of impulsivity on craving, relevant studies have shown that impulsivity could moderate the effect of social anxiety on alcohol craving, specifically, under the premise of increased impulsivity, socially anxious individuals receiving alcohol cues have a stronger craving for alcohol [69]. However, all the above studies were conducted on patients with alcohol use disorder as subjects. This study is a preliminary correlation analysis on the impact of impulsivity on substance craving, confirming that impulsivity does indeed have a positive impact on substance craving in substance users, and the mechanism of impulsivity on substance craving remains to be further explored in the future.

Moreover, alexithymia could influence relationships, such as relations with peers [70], doctor–patients relationship [71], which are critical to ameliorating substance craving [72]. According to the affective processing model of negative reinforcement [73], avoiding negative emotions is the priority motivation for individuals to maintain addictive behaviors. Substance users may avoid experiencing negative emotions by adopting more substance use behaviors. Impulsivity and alexithymia can evoke negative emotions, then increase substance craving, and eventually lead to substance use behaviors. In a word, exploring the role of impulsivity and alexithymia on substance craving has essential enlightenment and reference significance in the treatment of substance use behavior [74].

Second, the roles of aggressive behavior, positive psychological capital, interpersonal trust, and psychological security should be further investigated. Nevertheless, research on these factors and substance craving is rare so far. Thus, future studies can further explore the underlying influencing mechanism of these factors on substance craving.

### 4.3. Limitations and Future Directions

This study’s findings must be understood within the context of specific limitations. First, while the current study has looked at some of the psychosocial factors, some important variables, such as parenting style [75] and social support [76], should also be taken account into. Second, due to some force majeure factors (such as time, resources, etc.,), there were only male samples in our study, which makes it difficult to extend the results. Third, although we used machine learning to model the influencing factors of substance craving, this is only a preliminary inquiry, and more accurate data analysis methods may be needed to explore the influence degree or mechanism of each potential variable to better understand. Finally, we adopted a cross-sectional approach to collect data, which makes it hard to establish a true cause and effect relationship between these psychosocial variables and substance craving behavior among people with substance use. Therefore, in future studies, the combination of longitudinal data and behavioral experimentation should be applied.

## 5. Conclusions

The current study demonstrated that social factors, as well as psychological factors, play an important role in substance craving. Such information would be of great value to those professionals who help substance users develop recovery plans.

## Data Availability

The data that support the findings of this study are available from the corresponding author, upon reasonable request.
